# Identification and Characterization of *CYC*-Like Genes in Regulation of Ray Floret Development in *Chrysanthemum morifolium*

**DOI:** 10.3389/fpls.2016.01633

**Published:** 2016-11-07

**Authors:** Di Huang, Xiaowei Li, Ming Sun, Tengxun Zhang, Huitang Pan, Tangren Cheng, Jia Wang, Qixiang Zhang

**Affiliations:** Beijing Key Laboratory of Ornamental Plants Germplasm Innovation and Molecular Breeding, National Engineering Research Center for Floriculture, Beijing Laboratory of Urban and Rural Ecological Environment, Key Laboratory of Genetics and Breeding in Forest Trees and Ornamental Plants of Ministry of Education, School of Landscape Architecture, Beijing Forestry UniversityBeijing, China

**Keywords:** *Chrysanthemum morifolium*, *CYCLOIDEA*, TCP, inflorescence, flower symmetry, flower development

## Abstract

*Chrysanthemum morifolium*, one of the most economically important ornamental crops worldwide, is well-known for the elaborate and complex inflorescence which is composed of both bilaterally symmetrical ray florets and radially symmetrical disc florets. Despite continuing efforts, the molecular mechanisms underlying regulation of the two flower types are still unclear so far. *CYC*-like proteins have been shown to control flower symmetry or regulate flower-type identity in several angiosperm plant lineages. In this study, we conducted comparative analysis of the *CmCYC2* genes in two chrysanthemum cultivars and their F1 progenies with various whorls of ray florets. Six *CmCYC* genes were identified and sequenced, all of which were grouped into the CYC2 subclade. All the six *CmCYC2* genes were predominantly expressed in reproductive organs, and in particular in the petal of ray florets. Of these genes, the transcription level of *CmCYC2c* was highly up-regulated in ray florets of the double-ray flowered heads. In addition, the result that *CmCYC2c* was highly expressed at key developing stages indicates its role in regulating petal development. Furthermore, overexpression of *CmCYC2c* in *C. lavandulifolium*, one of the original species of *C. morifolium*, led to significant increase in flower numbers and petal ligule length of ray florets. Besides *CmCYC2c*, the expression of *CmCYC2f* was also significantly up-regulated in transgenic lines, implying a possible role in regulating development of ray florets. Both results of expression patterns and transgenic phenotypes suggest that *CmCYC2c* is involved in regulating ray floret identity in the chrysanthemum. This study will be useful for genetic manipulation of flower shape in chrysanthemum and hence promote the process of molecular breeding.

## Introduction

**FIGURE 1:**
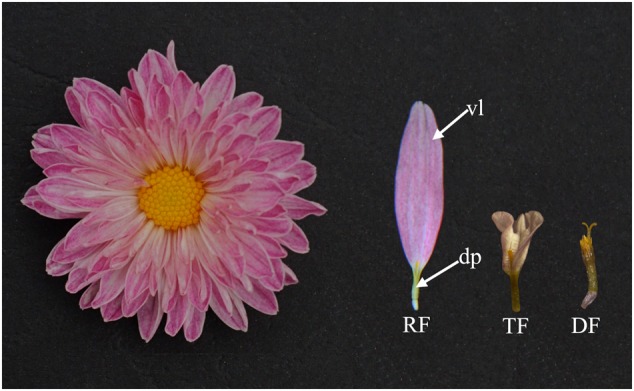
**Inflorescence of *Chrysanthemum morifolium*.** The marginal ray florets (RF) are female and bilaterally symmetrical. The central disc florets (DF) are hermaphrodite and bear carpel and pollen-producing stamens. The ray florets have showy ventral ligule (vl) formed by three fused petals while the two dorsal petals (dp) are rudimentary. Some chrysanthemum cultivars have intermediate *trans* florets (TF) similar to ray florets but with smaller or abnormal petal ligule.

Asteraceae is characterized for a highly compressed capitulum which superficially resembles a large single flower but is composed of numerous individual flowers (Funk, 2009). These flowers are divided into two types: ray and disc florets (DF; **Figure 1**). The peripherally located ray florets are bilaterally symmetrical with two rudimentary dorsal petals (dp) and elongated ventral ligule (vl) formed by three fused petals. Instead, the central DF are radially symmetrical with five equivalent petals (Bremer and Anderberg, 1994). The ray florets are generally female with arrested stamens, whereas the DF develop pollen-producing stamens in addition to carpels (Bremer and Anderberg, 1994; Gillies et al., 2002). Besides ray and DF, some chrysanthemum cultivars have a third flower type: *trans* floret (TF) which is morphologically similar to ray floret but with smaller or abnormal ventral petal ligule (**Figure 1**). Different combinations of these florets and variation of the petal types give rise to a variety of flower head types. Moreover, the presence of the showy ray florets in the capitulum has been shown to be associated with pollinator-mediated speciation, outcrossing rage and genetic diversity, and may lead to the evolutionary success of the Asteraceae (Marshall and Abbott, 1984; Sun and Ganders, 1990; Endress, 1999; Sargent, 2004; Juntheikki-Palovaara et al., 2014). Both classical and modern molecular genetic studies have indicated that the presence or absence of ray florets is mainly under the control of one or two major genes and some other modifier genes (Gillies et al., 2002; Andersson, 2008).

The TCP family of transcription factors has been shown to participate in regulating floral symmetry in a range of species (Luo et al., 1996, 1999; Cubas et al., 1999b, 2001; Kim et al., 2008; Yang et al., 2012). Proteins encoded by members of the TCP family share a conserved basic helix-loop-helix TCP domain (Cubas et al., 1999a; Aguilar-Martínez et al., 2007). The TCPs are divided into two classes: PCF and CYC/TB1. The CYC/TB1 lineage is specific of angiosperms and before the radiation of the core eudicots, it has experienced gene duplications, which give rise to three subclades, CYC1, CYC2, and CYC3 (Howarth and Donoghue, 2006). The studies in *Antirrhinum majus* show that the two CYC2 clade genes *CYC* and its paralog *DICHOTOMA* (*DICH*) are involved in flower symmetry regulation via specific expression in the dorsal petals of the flower and arresting the development of the dorsalmost stamen (Luo et al., 1996, 1999; Cubas et al., 1999b; Costa et al., 2005). The peloric radial symmetrical mutants of both *Linaria vulgaris* and *A. majus* are caused by the loss of expression of *LvCYC* and *CYC* via extensive DNA methylation or transposon insertion, respectively (Luo et al., 1996; Cubas et al., 1999b). Increasing evidences indicate that members of the CYC2 subclade have been repeatedly recruited to function in the control of floral zygomorphy during evolution. Similar to the adaxialized mutant in *Antirrhinum* caused by ectopic expression of *CYC*, the radial flowers of *Cadia purpurea* are dorsalized and all petals have acquired dorsal identity as a result of *LegCYC* expression in all five petals (Citerne et al., 2006). Meanwhile, *LjCYC2* from *Lotus japonicas* has also been found to be important in the establishment of dorsal identity (Feng et al., 2006). Furthermore, studies in *Arabidopsis* and *Primulina heterotricha* suggest that the persistent expression of *CYC*-like genes in later developmental stages is important for the development of corolla zygomorphy (Cubas et al., 2001; Yang et al., 2012). For example in *P. heterotricha*, two CYC2 clade proteins, *CYC1C* and *CYC1D*, have been shown to positively auto-regulate themselves and cross-regulate each other to maintain dorsally restricted gene expression and dorso-ventral differentiation in zygomorphic flowers (Yang et al., 2012). Therefore, the alteration of flower symmetry and modification of flower morphology (mainly for the dorsal petals) are usually caused by the spatial–temporal expression variation of the CYC2 subclade genes (Yang et al., 2015).

An independently CYC2-mediated pathway has apparently been recruited for inflorescence development in Asteraceae. Instead of mainly expressed in the dorsal portion to establish bilateral symmetry of individual flower, *CYC*-like proteins in Asteraceae participate in regulating the identity of flower types in inflorescence (Broholm et al., 2008; Kim et al., 2008; Juntheikki-Palovaara et al., 2014). In sunflower, the *turf* or *tub* mutants are characterized by a shift from zygomorphic to actinomorphic ray floret, due to insertion of transposable elements in the *HaCYC2c* TCP motif that leads to a premature stop codon (Fambrini et al., 2011, 2014a; Chapman et al., 2012). In contrast, in the *dbl* or *chry* mutants, caused by an insertion upstream the coding region, *HaCYC2c* is expressed throughout the flower head, converting DF into ray-like ones (Chapman et al., 2012). However, phenotypic analysis in F_2_ and F_3_ progenies derived from the crosses *Chry2* × *turf* demonstrates that the CACTA insertion is not always sufficient to change the expression of *HaCYC2c* gene and produce *Chry2* phenotype (Fambrini et al., 2014b). Ectopic expression of *Gerbera CYC*-like genes in transgenic *Gerbera* leads to similar changes with elongated petals and disrupted stamen development in DF (Broholm et al., 2008). Furthermore, it is possible that the context-specific protein complex involving *GhCYC2* proteins and their co-regulators may target different downstream genes (Juntheikki-Palovaara et al., 2014). Unlike in *Gerbera, RAY1, RAY2*, and *RAY3* in *Senecio* are only involved in promoting ventral identity in ray florets, whereas no change is found in DF (Kim et al., 2008; Chapman and Abbott, 2010; Garcês et al., 2016). Therefore, although a great amount of data have shown that these *CYC*-like genes in Asteraceae are key regulators in regulating flower development, a much more complex regulatory system seems to stay behind the complex flower head (Fambrini et al., 2014b; Juntheikki-Palovaara et al., 2014).

*Chrysanthemum morifolium*, one of the 10 most popular Chinese traditional flowers, is a typical example of radiate species with inflorescence composed of both zygomorphic and actinomorphic symmetric flowers (Teixeira da Silva, 2003; Teixeira da Silva et al., 2013; Qi et al., 2016). The contemporary chrysanthemum cultivars are characterized by the substantial variation in petal types and the complicated ploidy levels (from 2n = 4x = 36, to 6*n* = 54, 72, up to 90) (Liu et al., 2012). The allopolyploid and self-incompatible traits of chrysanthemum make it difficult to investigate the mechanism underlying regulation of the complex inflorescence. Recently, *CYC*-like TCP domain proteins have been shown to regulate morphological novelties during plant evolution (Luo et al., 1999; Kim et al., 2008; Hileman, 2014). In this study, two hexaploid chrysanthemum cultivars and several of their F1 progenies were selected to investigate the possible role of the chrysanthemum *CYC*-like genes in establishing the complex inflorescences. A total of six CYC2 genes were amplified from *C. morifolium*. The expression patterns of these genes were then compared during early inflorescence development stages and among several different floral heads with various whorls of ray florets. The function of *CmCYC2c*, which was remarkably up-regulated in the double-ray flowered heads (with multiple whorls of ray florets), was further characterized via overexpression in a wild diploid specie *C. lavandulifolium*. The growth of ray florets was significantly promoted in the positive transgenic lines. Meanwhile, the expression of *CmCYC2f* was also remarkably up-regulated. Hence, we speculate that the CYC2 subclade in chrysanthemum has expanded during evolution, and in particular, the *CmCYC2c* of these genes is capable of regulating the growth of ray florets.

## Materials and Methods

### Plant Materials and Growth Conditions

**FIGURE 2:**
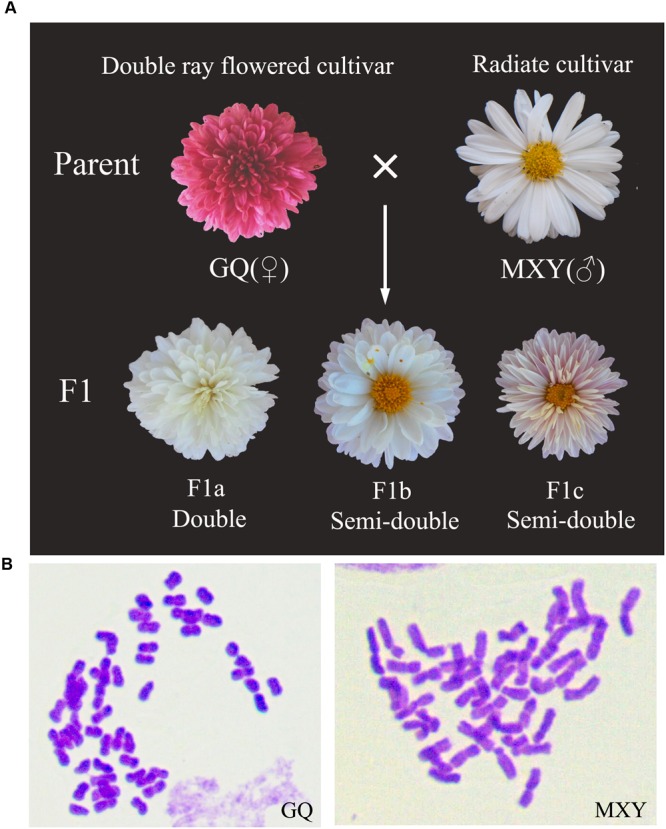
**Flower morphologies and chromosome numbers of several *C. morifolium* lines with various whorls of ray florets. (A)** Inflorescence of two chrysanthemum cultivars and their F1 progenies have intermediate whorls of ray florets compared with that of two parents. Cultivar abbreviations are as follows: GQ, *C. morifolium* ‘Guoqing xiaoliuhao’; MXY, *C. morifolium* ‘Mao xiangyu.’ **(B)** Chromosome numbers of the two chrysanthemum parents (GQ and MXY, 2*n* = 6*x* = 54).

Two cultivars of *C. morifolium* and their F1 progenies (**Figure 2A**), as well as the wild diploid species *C. lavandulifolium* were grown in the standard experimental fields at Xiao Tang Shan, affiliated to Beijing Forestry University, Beijing, China. C. *morifolium* ‘Guoqing xiaoliuhao’ (GQ) and ‘Mao xiangyu’ (MXY) (**Figure 2B**) are two hexaploid (2*n* = 54) ground-cover chrysanthemum cultivars with different whorls of ray florets. *C. lavandulifolium* (2*n* = 18) used for plant transformation is one of the original species of *C. morifolium* with relatively simple genetic background. Wild-type (WT) and transgenic plantlets of *C. lavandulifolium* with six leaves were transplanted into flowerpots containing a mixture of turf and vermiculite substrate (V:V = 1:1). All these plantlets were managed routinely in the standard greenhouse under a 16-h light/8-h dark cycle for 150 days, and then an 8-h light/16-h dark cycle at 25 ± 2°C with ∼55% relative humidity.

### Isolation of CmCYC2 Subclade Genes

Total RNA was extracted from young inflorescences of *C. morifolium* ‘Guoqing xiaoliuhao,’ ‘Mao xiangyu,’ and *C. lavandulifolium* using the TRIzol reagent (Invitrogen, USA) according to the manufacturer’s instructions. The RNA was treated with RQ1 RNase-free DNase (Promega, USA) according to the manufacturer’s protocol to remove residual genomic DNA. The RNA integrity was analyzed by gel electrophoresis, and its concentrations were measured and equalized within sample sets. The first strand of cDNA was synthesized based on 2 μg of total RNA using the M-MLV reverse transcription system (Promega, Madison, WI, USA). The TCP and R domain factors of CYC2 subclade genes were amplified using one pair of degenerate primers designed corresponded to the amino acid sequences ASKTLDWL and RARARERT of available GenBank Asteraceae sequences (accession number: FJ356704.1, FJ356700.1, EU429303.1, EU429304.1, and EU088368-EU088372). PCR conditions were the following: 94°C for 30 s, 54°C for 30 s, and 72°C for 1 min for 30 cycles. Multiple clones were sequenced and analyzed in an attempt to find all the *CYC*-like paralogs. The full length cDNAs were amplified by using the SMART RACE cDNA amplification kit (Clontech, Japan) to carry out 3′ and 5′ RACE (rapid amplification of cDNA ends). All of the amplified products were sub-cloned into the pMD18-T vector (Takara, Japan) and transformed into *E. coli* DH5α for sequencing. All primers were listed in Supplementary Table S1. The mRNA coding sequences of these genes have been uploaded to the GenBank database (accession numbers KU595426-KU595431 and KX161379-KX161384).

### Sequence Alignment and Phylogenetic Analyses

Multiple alignment of CYC/TB1-like protein sequences from *C. morifolium* and the other selected eudicots species were performed using ClustalX 2.0 with default parameters (Larkin et al., 2007), and the BioEdit software (version 7.1) was used to edit the aligned sequences. Protein sequences between the TCP and R domains were selected to generate maximum likelihood (ML) tree using PhyML (Guindon et al., 2010) under the Jones-Taylor-Thornton (JTT) model with 100 bootstrap replicates. Numbers above branches indicate local bootstrap value (>50% support). Prediction of conserved motifs of *CmCYC2* clade genes was performed using the MEME online tool ^1^.

### Scanning Electron and Light Microscopy

The young developing flower heads of MXY at different stages were collected and fixed overnight in FAA, and then dehydrated through a gradient ethanol series into 100% after being dissected under a stereomicroscope ethanol. After being chemically dried by washing in a 2-Methyl-2-propanol series and air-dried under vacuum, the samples were mounted on aluminum stubs and sputter coated with gold. Photographs were taken by a Hitachi S-3400N scanning electron microscope (SEM) in Beijing Forestry University.

The flower buds in 100% ethanol (procedures before this were the same as that mentioned above for SEM) were moved into a gradient xylene series. Then, the samples were embedded in paraffin and cut into 8 μm sections. For histological staining, paraffin was removed with xylene and the sections were stained with safranin (1% in water) and fast green (0.3% in 95% ethanol). The sections were observed and photographed under a light microscope (Zeiss Axio scope. A1) in Beijing Forestry University.

### Quantitative Real-Time PCR Analyses

**FIGURE 3:**
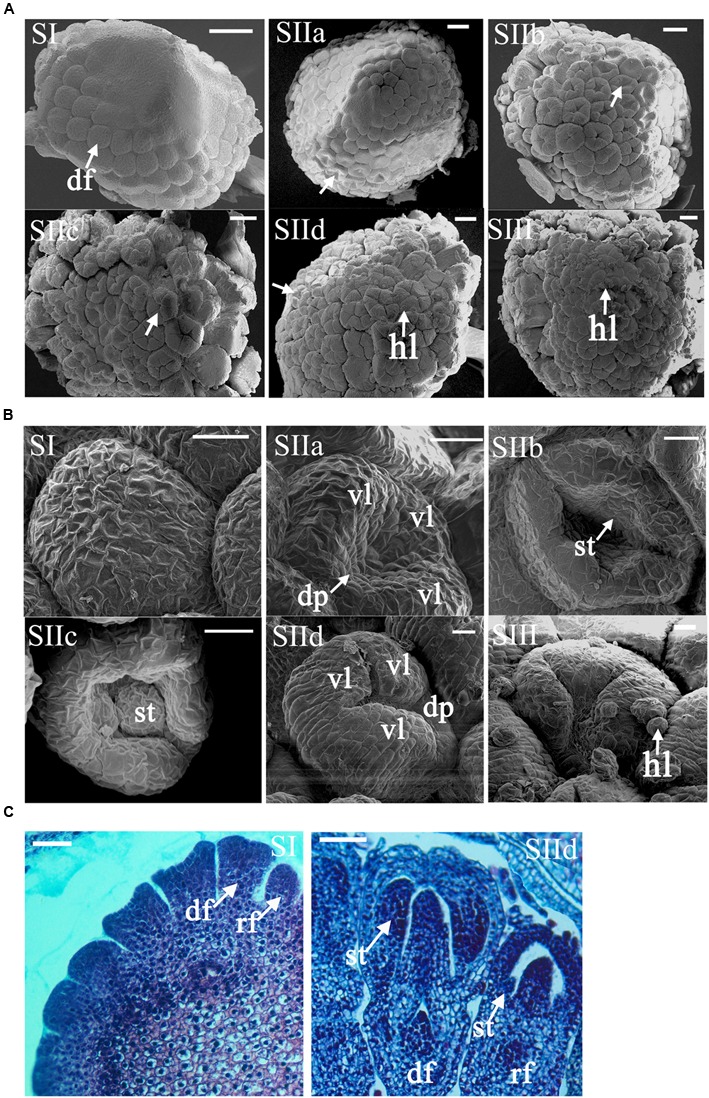
**Morphological analysis of the early development stages of ray and DF in *C. morifolium* ‘Mao xiangyu’ (MXY). (A)** SEM images show developing capitula (stages I–III). The primordia of the disc florets (df) develop acropetally from the basal to the upper during the whole ontogeny process. The ring-shaped petal primordia began to form at stage IIb (arrowed) for the inner whorls of DF, whereas the peripheral whorls of DF already had stamen primordia at stage IIa (arrowed). The petal of peripheral DF finally covered the inner organs at stage IIc (arrowed). At stage IId, nearly all the DF finished organ differentiation except for the innermost ones (arrowed). Hair like (hl) structures began to emerge at stage IId. Scale bars: 100 μm. **(B)** SEM images show developing ray florets (stages I–III). Asymmetry growth of petals in ray florets started to emerge at stage IIa with accelerated elongation of three ventral petal ligules (vl) and ceased growth of two dorsal petals (dp). The stamens and two dorsal petals gradually ceased growth while the ventral petal ligule continued to elongate during stage IIc-III. Scale bars: 20 μm. **(C)** Histological analysis of ray (rf) and disc floret (df) primordia at stage I and II d. The primordia of the ray florets (arrowed) appeared to be smaller than those of the disc ones at stage I. The development of rudimentary stamens (st) in ray florets lagged behind at stage IId. Scale bars: 50 μm.

Quantitative RT-PCR (qRT-PCR) assay was used to detect the expression levels of the *CmCYC2* genes in inflorescence primordia of MXY at six early developmental stages (stages I-III, **Figure 3**) and one late stage (stage IV) at which the petal ligules of ray florets began to expand (**Supplementary Figure S1**). To analyze the specific expression patterns of the *CmCYC2* genes in ray and DF of *C. morifolium*, the double-ray flowered cultivar GQ and the radiated cultivar MXY, as well as two of their F1 progenies (F1a and F1b) with intermediate whorls of ray florets were prepared (**Figure 2A**). Each sample was a pooling of six to eight inflorescences at later developing stages (pooled from stage 3, 4, 6, 7, 8, and 9) (**Supplementary Figure S1**). We defined the later inflorescence developmental stages comparable to those defined in *Gerbera* (Bradley et al., 1993; Laitinen et al., 2007). In addition, the disc floret samples were only excised from their centermost florets while the ray floret samples were a mix of flowers from every whorl. For tissue-specific expression analysis, vegetative and reproductive organs (a mixture of stage 8 and 9) were dissected from another F1 progeny (F1c) which developed some *trans*-like florets between the normal ray and disc ones (**Figure 2A**). Additionally, the stamen sample was pooled from DF only, and the ovary plus stigma and style sample was a mixture of all the three types of florets. All plant materials collected were immediately placed in liquid nitrogen and stored at -80°C until extraction. Three biological replicates were collected for each sample. The extraction of total RNA and the synthesis of the cDNA were performed as described above. The cDNA sample was diluted five times with 80 μl of deionized water for all gene expression analyses. The qPCR reactions were performed using a Mini Opticon Real-time PCR System (Bio-Rad, USA) with SYBR Premix Ex TaqII kit (TaKaRa, Japan) following the manual’s recommendations. All gene-specific primer pairs for qRT-PCR were listed in Supplementary Table S1, and their amplification efficiencies were analyzed to make sure that they were closed to 100%. The PCR program was conducted with an initial step of 30 s at 95°C, followed by 40 cycles at 95°C for 5 s, 58°C for 30 s, and 72°C for 30 s. Product specificity for each primer pair was verified by melting-curve analysis. The relative expression levels were calculated using the 2^-ΔΔ^Ct method, with *CnActin* (GenBank accession number KF305683.1) gene as the endogenous control.

### Vector Construction and Plant Transformation

The ORF of *CmCYC2c* was obtained by PCR amplification using gene-specific primers *CmCYC2c-*F2 and *CmCYC2c-*R2 (both containing NcoI enzyme site). Driven under the control of the CaMV 35S promoter, the *CmCYC2c* was cloned into a modified binary pCOMBIA1304 vector. Then the 35S::*CmCYC2c* plasmid was transformed into *C. lavandulifolium* by the *Agrobacterium tumefaciens* EHA105-mediated freeze-thaw method (Chen et al., 2004). For transformation, small leaf pieces with a diameter less than 8 mm were cut from newly formed expanding leaves of *C. lavandulifolium* using scalpels. After 48 h pre-culture on MS medium, these leaves were soaked in the *Agrobacterium* bacteria solution containing 20 μM acetosyringone (final OD_600_ = 0.4–0.6) for 10 min, with excess broth sucked away by sterilized absorbent paper. Then the leaf pieces were cultured on the CI (callus-induction) medium (MS medium +1.0 mg l^-1^ 6-BA, 0.5 mg l^-1^ NAA) for 3 days in darkness. After being washed with sterilized water, the explants were placed on the bacteria elimination medium (CI medium containing 400 mg l^-1^ carbenicillin) for 5 days, and then were transferred to the selection medium I (CI medium containing 400 mg l^-1^ carbenicillin and 8 mg l^-1^ hygromycin) for the selection of putatively transformed calli. After two subcultures, the explants were placed on the selection medium II (containing only 200 mg l^-1^ carbenicillin) to promote callus differentiation. The medium was replaced every 2 weeks until the regenerated plantlets grow up with 2–4 leaves. Then the putatively transformed plantlets were transferred to the rooting medium (MS medium + 10 mg l^-1^ hygromycin, 200 mg l^-1^ carbenicillin). All the materials were cultured at the same conditions with 16 h light/8 h dark at 24 ± 2°C. The transgenic plants selected from hygromycin were verified using a pair of CaMV 35S promoter-specific forward primer and *CmCYC2c*-specific reverse primer (Supplementary Table S1).

### Gene Expression Analysis and Phenotypic Observation of Transgenic Lines

RNA extraction and cDNA synthesis were performed as described above from full opened inflorescence. We used two primer pairs to distinguish the expression levels of the transgene (*CmCYC2c*) and the endogenous gene (*ClCYC2c*) in full opened inflorescences of six positive transgenic lines. One specific primer pair *CmCYC2c*-F1/*CmCYC2c*-R1 was designed corresponded to the ORF sequence of the transferred gene *CmCYC2c*, the other pair *ClCYC2c*-utr-F1/*ClCYC2c*-utr-R1 was designed according to the 3′ UTR sequence of endogenous *ClCYC2c*. The ray florets of the three positive lines, showing obvious phenotypic changes, were used to detect the expression levels of all the six *ClCYC2* clade genes. The *ClActin* gene (GenBank accession number JN638568.1) was used as an internal control. All primers were shown in Supplementary Table S1. Both WT plants and transgenic lines expressing the empty vector were used as the negative control plants.

The phenotypes of transgenic lines were observed and recorded during the development process. The numbers of ray and DF were calculated from the same inflorescence using fifteen independent flower heads per line. In addition, the petal ligule lengths of ray florets were measured from 15 inflorescences of each line. The top three fully open inflorescences were sampled from five lateral branches, the second one below each shoot apex, of both positive transgenic lines and control lines. Statistical testing was done with SPSS17.0 (SPSS Inc., Chicago, IL, USA) using one-way ANOVA and Duncan LSD multiple comparisons tests.

## Results

### Ontogenesis of Early Flower Primordia of *C. morifolium* ‘Mao xiangyu’

The capitulum of MXY contains 8–9 whorls of DF and two whorls of peripheral ray florets (**Figure 2A**). To better understand the morphologic traits of ray and DF of MXY, we normalized the development process into four phases: (I) initiation of floral primordia; (II) differentiation of floral organs; (III) growth of floral organs; and (IV) maturation of inflorescence (data not shown as the capitulum of this stage is big enough to be clearly observed with naked eye). The sizes of the capitula corresponding to each stage were shown in Supplementary Table S2. At stage I, the differentiation of inflorescence primordia was completed and individual flower primordia (arrowed) arose acropetally through the capitulum (**Figures 3A,C**, SI). The ray and disc floret primordia were indistinguishable undifferentiated bumps at this stage (**Figures 3A,B**, SI). At stage IIa, the marginal four whorls of DF began to form petal and stamen primordia (arrowed) while the inner four whorls were still undifferentiated bumps (**Figure 3A**, SIIa). From stage IIb to IIc, the differentiation of floral organ was continuing, those undifferentiated innermost bumps gradually turned into ring-shaped petal primordia (arrowed) with recognizable raised stamen primordia (**Figure 3A**, SIIb). Additionally, the petal lobes of peripheral DF continued to elongate and finally covered the developing stamen and carpel primordia (arrowed, **Figure 3A**, SIIc). The floral organ differentiation of ray florets seemed to lag behind the outermost DF at stage IIa, which became noticeable at stage IIb when the stamen primordia (st, arrowed) appeared in ray florets (**Figure 3B**, SIIa and SIIb). In ray florets, the zygomorphy of five petal ligules appeared (arrowed) soon after the petal primordia occurred at stage IIa. Further, the asymmetry became prominent with accelerated expanding of three fused ventral ligules (vl) and ceased growth of two dorsal petals (dp) when the stamen primordia kept on developing (**Figure 3B**, SIIa-SIIc). At stage IId, nearly all ray and DF finished their organ differentiation except for the innermost DF (arrowed, **Figure 3A**, SIId). The marginal ray florets eventually developed into female with arrested growth of stamens at stage IId, whereas the DF were fully bisexual (**Figure 3C**, SIId). Furthermore, the hair-like structures (hl) began to emerge on the surface of the peripheral flowers (**Figure 3A**, SIId). At stage III, the differentiation of floral organs was completed, and all flowers continued to grow and undertook further development to bloom (**Figures 3A,B**, stage III). In addition, all flowers were attached with those hair-like structures.

### Sequence Analysis of the Chrysanthemum *CYC*-Like Homologs

**FIGURE 4:**
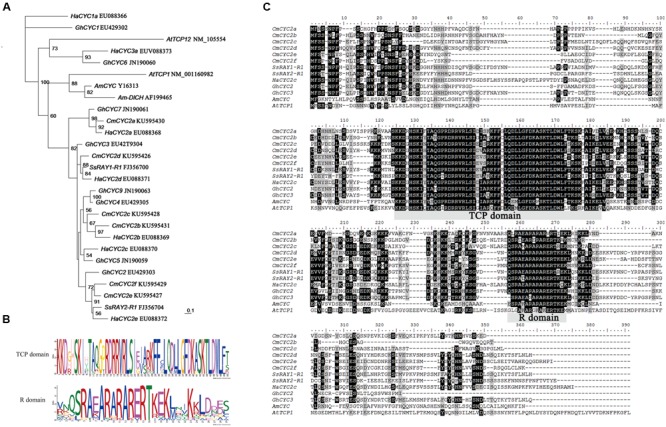
**Multiple alignment and phylogenetic analysis of CYC-like proteins from Asteraceae and selected other species. (A)** Phylogenetic analysis of selected CYC/TB1-like proteins using the maximum likelihood method. Local bootstrap probabilities are indicated for branches with >50% support. The accession numbers of all listed sequences retrieved from GenBank database are shown following gene names. Species abbreviations in gene names are as follows: Am, *Antirrhinum majus*; At, *Arabidopsis thaliana*; Gh, *Gerbera hybrid*; Ha, *Helianthus annuus*; Cm, *Chrysanthemum morifolium*; Ss, *Senecio squalidus*. **(B)** The two conserved TCP and R domains are analyzed by MEME software. The overall height of the stack indicates the sequence conservation at that position. **(C)** Alignment of deduced amino acid sequences of *CmCYC2* and *CYC*-like proteins from other organisms. Identical amino acid residues are shaded in black, similar in gray.

To study the role of CYC2 subclade transcription factors in establishment and evolution of the complex flower head of chrysanthemum, six genes encoding these proteins were isolated from young developing inflorescences by an extensive screening of putative homologs from PCR products. These genes were named according to a previously published nomenclature and are as follows: *CmCYC2a, CmCYC2b, CmCYC2c, CmCYC2d, CmCYC2e*, and *CmCYC2f* (GenBank accession nos. KU595426-KU595431). Excluding introns, the length of each gene varied from 774 to 969 bp (258 to 323 inferred amino acids). A conserved motif analysis showed that all these genes contain the conserved TCP and R domain typical for the CYC2/ECE subfamily (**Figure 4B**). The sequence variation among these genes are mainly located within the non-conserved regions (**Figure 4C**).

We performed phylogenetic analysis on a selected set of TCP class II proteins (between the TCP and R domains) from various plant species to explore the evolutionary relationships and diversification of the *CmCYC2* subclade genes. The maximum likelihood analysis placed all the six *CmCYC2* genes in CYC2 clade together with all the other listed Asteraceae sequences (**Figure 4A**). Therefore, our focus here was on the CYC2 subfamily. The most clearly supported orthologous were *CmCYC2a* and *HaCYC2a* along with *GhCYC7*, as well as the recently duplicated gene pair *CmCYC2b* and *HaCYC2b* grouping with *CmCYC2c*. Although the relationship was not well supported, *CmCYC2f* and *CmCYC2e* were grouped into one clade, and sister to a *Senecio* ray floret specific gene *SsRAY2*. The *CmCYC2d* gene was clustered with the other *Senecio* ray floret specific gene *SsRAY1*. In conclusion, *CmCYC2f* and *CmCYC2e* showed a sister relationship whereas the other four *CmCYC2* genes were sister to the other Asteraceae sequences, suggesting both lineage-specific and shared duplications in Asteraceae.

### Expression Patterns of the Chrysanthemum *CYC*-Like Homologs

**FIGURE 5:**
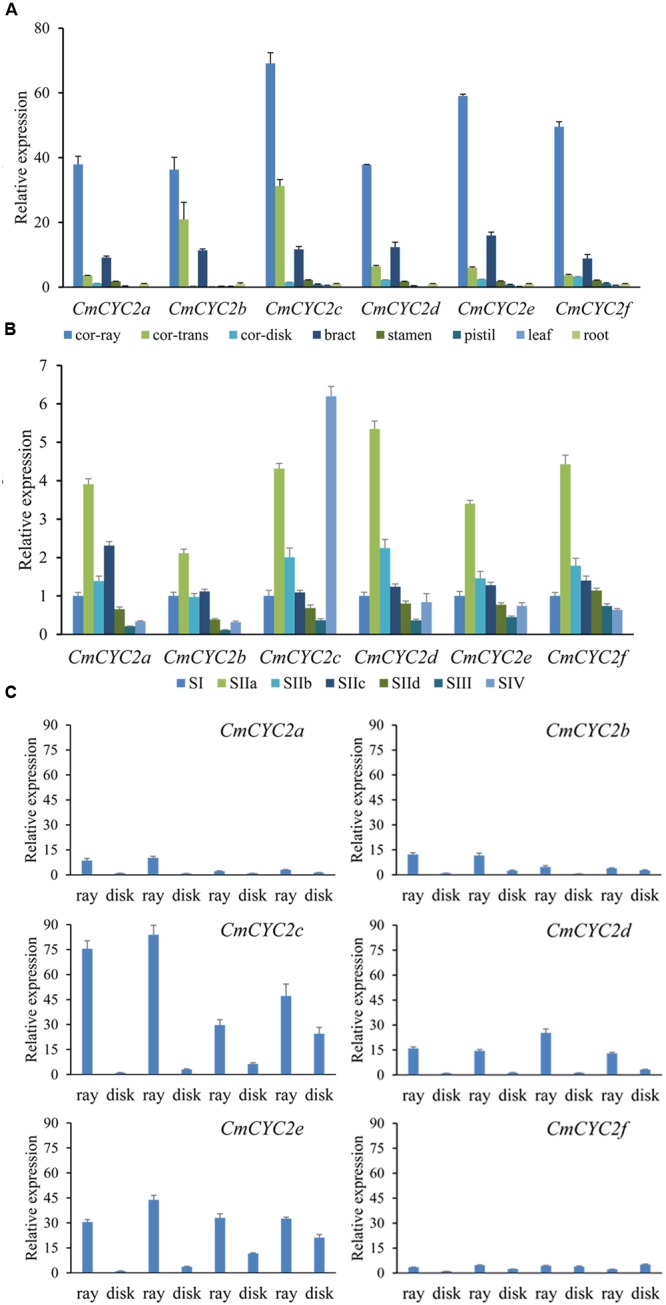
**The relative expression levels of the six chrysanthemum *CYC2* genes.** The columns show average expression levels of three biological replicates for each sample. Expression levels of *CmActin* were used for normalization. **(A)** Expression patterns of *CmCYC2* genes in different tissues of F1c (a F1 progeny). Tissues analyzed including: corolla of ray florets (Cr), corolla of *trans* florets (Ct), corolla of disc florets (Cd), involucral bract (Br), pistil (Pi) including ovary, stigma and style, stamen (St), leaf and root. **(B)** Expression patterns of *CmCYC2* genes in flower buds of *C. morifolium* ‘Mao xiangyu’ (MXY) during inflorescence development. SI–SIV indicate development stages (stage1–stage IV) of inflorescence. **(C)** Relative expression analysis of the *CmCYC2* subclade genes in two chrysanthemum cultivars and their F1 progenies have intermediate whorls of ray florets. RNA was isolated from the ray florets (ray) and DF (disc) of four types of flower heads. The values were normalized to the *CmActin*, and were graphically scaled to the disc floret of GQ, with error bars depicting the standard deviation of three biological replicates.

According to the earlier results in *Senecio, Gerbera* and *Helianthus* (Broholm et al., 2008; Kim et al., 2008; Fambrini et al., 2011; Tahtiharju et al., 2012), CYC2 subclade genes are involved in regulating the complex inflorescence architecture of Asteraceae. To look into the possible role of the *CYC*-like genes in chrysanthemum, we investigated the transcription levels of the six *CmCYC2* genes in different tissues of F1c (**Figure 5A**). All the six *CmCYC2* genes were mainly expressed in floral reproductive organs, although they were weakly expressed in vegetative organs, in accordance with the results in *Gerbera* and sunflower (Broholm et al., 2008). In addition, all these genes were found to be predominantly expressed in petal of ray florets and barely expressed in that of DF. *CmCYC2b* and *CmCYC2c* in contrast were strongly expressed in petal of middle *trans*-like florets, which was not generally true for other *CmCYC2* genes. Contrary to the previous studies in other species (Broholm et al., 2008; Tahtiharju et al., 2012), all the *CmCYC2* genes were surprisingly relatively highly expressed in the involucrate bracts while slightly expressed in stamens and pistil (including the ovary, styles, and stigma). qRT-PCR reactions were also performed to compare the expression patterns of these genes in flower buds of MXY at both early and late development stages, as shown in **Figure 5B**. Interestingly, the expression of these genes all peaked at stage IIa and then decreased gradually and maintained at relative low levels, except for *CmCYC2c*, which was remarkably up-regulated at stage IV (mature flower buds).

To further investigate any flower-type specific role of the *CYC*-like genes, we compared the expression patterns of these genes in two chrysanthemum cultivars and two of their F1 progenies (**Figures 2A** and **5C**). Similar to the previous studies (Broholm et al., 2008; Tahtiharju et al., 2012), all genes were found to be expressed at much higher levels in ray florets than in DF, as shown in **Figure 5C**, except that *CmCYC2f* was expressed at more or less comparable levels in all florets. *CmCYC2d* and *CmCYC2e* were expressed at generally similar levels in all of the tested ray florets, and did not display phenotype difference among four types of flower heads. In contrast, the expression of *CmCYC2c* was dramatically up-regulated in ray florets of the double-ray flowered heads (GQ and F1a), compared with the expression in the radiate (MXY) and semi-double ray flowered (F1b) heads. Although *CmCYC2a* and *CmCYC2b* exhibited an expression pattern similar to the *CmCYC2c*, they were expressed at much lower levels.

In short, *CmCYC2c* was abundantly expressed at two key stages of petal development in inflorescence primordia, and in petals of both ray and *trans*-like florets. Moreover, in a series of flower heads with various whorls of ray florets, the transcript levels of *CmCYC2c* were significantly up-regulated in ray florets of the double-ray flowered heads. We therefore conclude that *CmCYC2c* is a strong candidate as a key regulator of ray floret identity in chrysanthemum, and hence need a further verification through transgene.

### Transgenic Analysis of *CmCYC2c* in *C. lavandulifolium*

**FIGURE 6:**
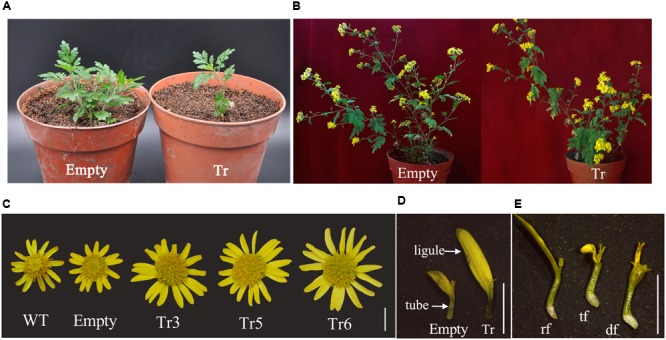
**Phenotype of the transgenic plants overexpression *CmCYC2c*. (A)** The vegetative growth of the positive overexpression line (Tr) was retarded at seedling stage compared with that of the line with empty vector (Empty). **(B)** Phenotype of the blooming plants. **(C)** The effect of activated *CmCYC2c* function on the inflorescence. The capitula of positive transgenice lines appear to be larger due to enhanced length of ventral petal ligule in ray florets and increased number of ray florets per head. **(D)** The ventral petal ligule of ray florets in positive transgenic lines are longer than those in lines with empty vector. **(E)** *Trans*-like flowers (tf) are generated between the normal ray (rf) and disc florets (df) in one transgenic line. (Scale bars: 5 mm)

According to our results, *CmCYC2c* was predominantly expressed in ray and *trans*-like florets (if presented) of the double-ray flowered inflorescence. Thus, the ORF of *CmCYC2c* was transformed under the CaMV 35s promoter into *C. lavandulifolium*. Finally, three positive lines showing obvious phenotypic changes were chosen for further analysis. Ectopic expression of 35S::*CmCYC2c* retarded the vegetative growth and outgrowth of lateral branches in young plantlets (**Figure 6A**). However, the growth of these young seedlings restored gradually later on and they produced inflorescence finally (**Figure 6B**). Further, the capitula appeared larger in the over-expression lines due to increased flower number and elongated petal ligule length of ray florets, compared with the WT plants and transgenic plants with empty vector (**Figure 6C**). The average length of petal ligule in ray florets was remarkably increased from 4.72 mm in empty vector to 6.12–7.04 mm in positive transgenic lines (**Figure 6D**; **Table 1**). In addition, the average number of ray florets per inflorescence was significantly enhanced from 13.8 in empty vector to 16–18 in positive transgenic lines (**Table 1**). Furthermore, we found some *trans*-like florets in one of the transgenic lines, which were morphologically similar to ray florets but with short petals and abnormal stamens (**Figure 6E**). In some inflorescences, the growth of ray florets was severally inhibited and resulted in malformation (data not shown). Interestingly, no phenotypic difference was observed in DF among different lines, in accordance with the results in *Senecio* (Kim et al., 2008; Garcês et al., 2016).

**Table 1:** Ray flower traits affected in the *CmCYC2c* transgenic lines in *C. lavandulifolium.*

Genotype	Ligule length of ray florets (mm)	Number of ray florets per capitulum
Wild-type(WT)	4.89 ± 0.49a	13.75 ± 1.37a
35S::Empty vector	4.72 ± 0.41a	13.8 ± 1a
35S:: *CmCYC2c*-TR3	6.48 ± 0.41c	16.15 ± 1.27b
35S:: *CmCYC2c*-TR5	6.12 ± 0.27b	18.1 ± 1.55c
35S:: *CmCYC2c*-TR6	7.04 ± 0.71d	16.2 ± 1.36b

*Mean values ± SD are analyzed with a one-way ANOVA. Different letters adjacent to each value in the same column indicate significant differences at *P* < 0.05 by Duncan LSD test. The length of the petal ligule of ray florets and the number of ray florets per capitulum were documented from three independent transgenic lines in comparison with WT plants and transgenic plants with empty vector.*

**FIGURE 7:**
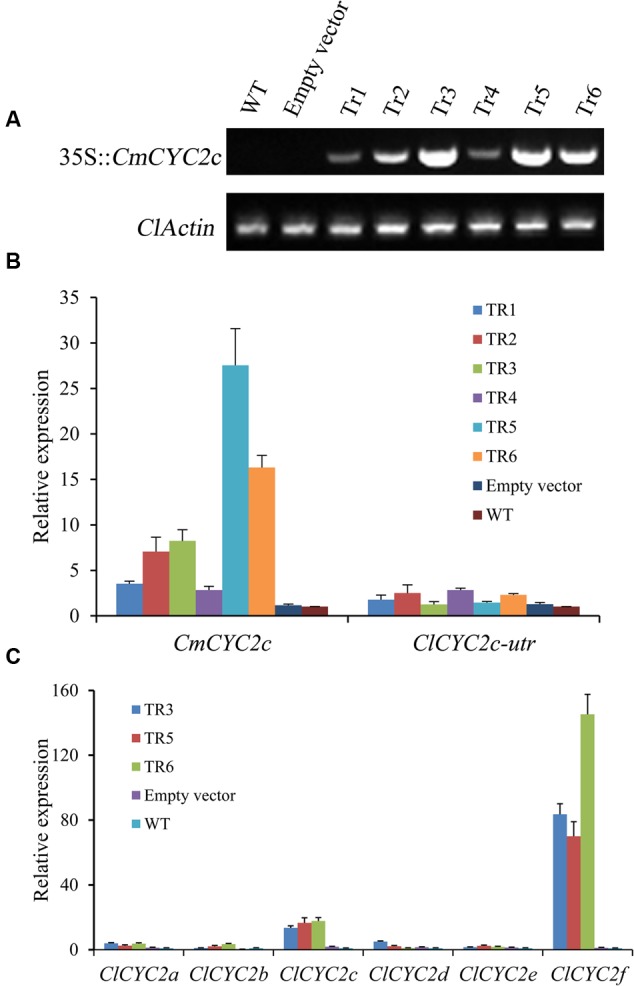
**Transcription levels of the CYC2 subclade genes in activated *CmCYC2c* lines, wild-type plants (WT) and transgenic plants with empty vector (empty vector). (A)** Examination of 35S::*CmCYC2c* expression in inflorescences of the positive transgenic lines and both control plants, and *ClActin* gene was used as a positive control. **(B)** The expression levels of the transferred *CmCYC2c* gene and endogenous *ClCYC2c* gene in inflorescences of the transgenic plants and both control lines. **(C)** qRT-PCR assay was used to determine the expression levels of the six *ClCYC2* genes in ray florets of the selected transgenic lines and both control lines. The columns show average value with SD bar from three biological replicates. Several *CmCYC2c* overexpressing lines (Tr1–Tr6), WT plants and transgenic plants carrying the empty vector were used for qRT-PCR assay.

Moreover, the six independent positive transgenic lines that expressed *CmCYC2c* constitutively (**Figure 7A**) were further confirmed by qRT-PCR assay (**Figure 7B**). The expression of *CmCYC2c* was highly up-regulated in positive lines when compared with that of the endogenous *ClCYC2c* gene. Furthermore, given that the CYC2 clade proteins have been shown to cross-regulate each other to positive regulate gene expression (Tahtiharju et al., 2012; Yang et al., 2012), the effect of *CmCYC2c* on the other five *ClCYC2* genes was also analyzed by qRT-PCR assay (**Figure 7C**). The results showed that, besides a significant up-regulation for *ClCYC2c*, the expression of *ClCYC2f* was also strongly induced in ray florets of the transgenic lines when comparing with that in both control ray florets. No obvious difference was found for the expression levels of the other four *ClCYC2* genes among these lines.

## Discussion

### Duplication and Divergence of Chrysanthemum *CYC*-Like Genes

*CYC*-like genes in Asteraceae have been shown to function as key regulators controlling the differentiation of the specific flower type (Marshall and Abbott, 1984; Broholm et al., 2008; Kim et al., 2008; Fambrini et al., 2014b). Much of the work has focused on the three model species: *S. vulgaris* (Kim et al., 2008; Garcês et al., 2016), *H. annuus* (Chapman et al., 2008, 2012) and *G. hybrid* (Broholm et al., 2008; Juntheikki-Palovaara et al., 2014). Surprisingly, few reports have been published on the function of these genes in the chrysanthemum, which is characterized by the extreme diversity of floral morphology. It has been previously reported that the CYC2 subclade has undergone the greatest number of gene duplications within the Asteraceae, such as five *CYC*-like genes in *H. annuus* and six in *Gerbera* (Broholm et al., 2008). No more copies have previously been reported in any other plant lineages studied thus far. Correspondingly, six *CmCYC2* genes were obtained in chrysanthemum with highly conserved TCP and R domains. Gene and whole-genome duplications in plants are known to increase genomic complexity and have been suggested to produce new genes that allow the evolution of functional novelty (Jiao et al., 2011). The duplication of CYC2 subclade in Asteraceae seems to support the idea that members of this subclade may have novel and important functions contributing to the complex inflorescence structure in Asteraceae. Intriguingly, the strong gene candidates in specifying flower zygomorphy in Asteraceae were grouped into different clades according to the phylogenetic trees reported previously by Chapman et al. (2012) and Tahtiharju et al. (2012) and the current study. For example the *HaCYC2c* in sunflower (Chapman et al., 2012), the *GhCYC2* in *Gerbera* (Tahtiharju et al., 2012), the *SsRAY1* and *SsRAY2* in *Senecio* (Kim et al., 2008*)*, and the *CmCYC2c* in chrysanthemum were placed into five different groups, rather than showing sister relationships. These results indicate that paralogs of this gene family have been independently recruited to regulate zygomorphy in different species within the Asteraceae. Moreover, similar to the reports in sunflower and *C. lavandulifolium* (Tahtiharju et al., 2012; Ren and Guo, 2015), ray florets could be easily distinguished from disc ones in chrysanthemum at early stage of flower primordia development. The reason is that the asymmetric growth of the five petals in ray florets occurred soon after the petal primordia emerged. In *Gerbera*, however, the ray and disc petal primordia do not show difference in growth until late developing stages (Laitinen et al., 2006; Tahtiharju et al., 2012). Thus, the morphological divergence within Asteraceae in this respect is also consistent with the organismal phylogeny. As *G. hybrida* (Mutisieae) is a phylogenetically more distant specie when compared with the other three species (Bremer and Anderberg, 1994; Panero and Funk, 2008). This explanation can be further supported by the phylogenetic analysis that all the *CmCYC2* genes were sister to the corresponding CYC2 members of sunflower and *Senecio*, whereas they were not clustered with most *Gerbera* CYC2 genes.

### Expression Analysis Indicates both Early and Late Functions for *CmCYC2c* during Ray Floret Development

Typically, the expression of CYC2 subclade genes is restricted to the dorsal regions of the flower, for example, in *A. majus* (Luo et al., 1999) and *Veronica montana* (Preston et al., 2009). In Asteraceae, however, the expression of *CYC*-like genes is excluded from the dorsal petals and has shifted to the ventral part of ray florets (Chapman et al., 2008; Kim et al., 2008; Tahtiharju et al., 2012; Juntheikki-Palovaara et al., 2014). For example, *RAY1, RAY2*, and *RAY3* in *Senecio*, proved to control ray versus disc floret identity, are expressed only in ray florets (Kim et al., 2008; Garcês et al., 2016). Here in chrysanthemum, the *CmCYC2* genes were also predominantly expressed in petals of ray florets, consistent with what has been reported previously in Asteraceae (Chapman et al., 2008; Tahtiharju et al., 2012). The appearance of *trans*-like florets in the F1 progeny (F1c) suggests it is a transition type between ray and DF. In addition to petals of ray florets, *CmCYC2c* and *CmCYC2b* were also abundantly expressed in petals of *trans*-like florets, suggesting their possible role in promoting the growth of ventral petal ligule. This fits well with our data in transgenic lines that over-expression of *CmCYC2c* leads to elongated ventral ligule length in ray florets. Similar results have been reported for *GhCYC4*, which also shows ray/TF specific expression, and function in enhancing the growth of petal ligules (Juntheikki-Palovaara et al., 2014). Detailed expression analysis in *Gerbera* show that *GhCYC2* is exclusively expressed in the ventral domain, and the expression of *GhCYC3, GhCYC4, GhCYC5*, and *GhCYC7* is detected both in the fused ventral ligule and two rudimentary dorsal petals.

The transcript levels of the six *CmCYC2* genes were analyzed in two chrysanthemum cultivars and their F1 progenies with intermediate whorls of ray florets. All these genes were predominantly expressed in ray florets, and most of them showed no or some expression variation across genotypes. However, the expression of *CmCYC2c* was significantly up-regulated in the double-ray flowered heads, indicating it is a strong candidate as a regulator of ray floret identity. It is supported by the fact that the 35s::*CmCYC2c* transgenic lines generate more ray florets per capitulum in comparison with that in the control lines. Despite well-known for the abundant flower shape, nearly all wild original crossing parents of modern chrysanthemum are characterized by multiple whorls of DF surround by a single whorl of peripheral ray florets. Of these wild original germplasm, *C. lavandulifolium* is one typical and vital diploid specie. The regulatory mechanism controlling the growth of ray florets in chrysanthemum is still not clear yet. Therefore, it is interesting and meaningful that the number and morphology of the ray florets of *C. lavandulifolium* are changed by ectopic activation of *CmCYC2c*. According to the reports by Tahtiharju et al. (2012), *HaCYC2c* and *GhCYC3* are indicated to be strong candidates in controlling of ray floret identity owing to their specific expression in ray flowers. In sunflower, the expression of *HaCYC2b* and *HaCYC2e* are generally similar across different genotypes, whereas increased *HaCYC2c* expression is detected across the inflorescence of *double-flowered* (*dbl*) mutant (with only ray florets) (Chapman et al., 2008, 2012). Furthermore, overexpression of *HaCYC2c* due to an insertion upstream of the start codon generates *dbl* mutant, providing functional evidence for its role in ray formation (Chapman et al., 2012). In addition, *GhCYC3* in *Gerbera* is highly up-regulated in the centermost ray floret primordia of the crested cultivar, which can convert DF into ray-like ones by promoting petal ligule growth in transgenic lines (Broholm et al., 2008).

The *CmCYC2* subclade genes also share strikingly similar expression patterns during the early inflorescence developing stages (from SI to SIII). All these genes were highly expressed at stage IIa and then decreased gradually. However, *CmCYC2c* was also highly expressed at stage IV when compared with the other five genes. Since the petal of ray florets begin to differentiate at stage IIa and further expand at stage IV, we speculate that *CmCYC2c* may function in regulating petal development at both early and late stages. Similar functions have been reported previously for *CYC* and *GhCYC3*, which are involved in modifying petal growth owing to cell expansion or proliferation at different stages of development (Luo et al., 1996; Juntheikki-Palovaara et al., 2014). However, further studies are required to elucidate the expression patterns of the *CmCYC2* genes at the organ and tissue level using, for example, RNA *in situ* hybridization.

### Overexpression of *CmCYC2c* in *C. lavandulifolium* Promotes the Petal Growth of Ray Florets

An increasing number of evidence demonstrate that *CYC*-like genes participate in controlling flower symmetry and typically the growth of the dorsal parts of a flower as shown, for example, in *A. majus* (Luo et al., 1996, 1999) and *I. amara* (Busch and Zachgo, 2007). In *Antirrhinum*, for example, *CYC* and *DICH* regulate flower symmetry by promoting dorsal petal growth but repressing dorsalmost stamen development (Luo et al., 1996, 1999). Similarly, in *Mohavea confertiflora*, the expression of *McCYC* and *McDICH* in both dorsal and lateral regions is correlated with the abortion of both dorsal and lateral stamens and the increase of dorsal petal sizes (Hileman and Baum, 2003). In addition to controlling symmetry in a single flower, the TCP domain regulatory proteins in Asteraceae have developed a novel role in regulation of floral identity within the capitulum (Broholm et al., 2008; Fambrini et al., 2014b; Juntheikki-Palovaara et al., 2014; Garcês et al., 2016). Abundant expression data have shown that these members of CYC2 subclade in Asteraceae play a pivotal role in promoting or repressing the growth of ventral petal ligule, and sometimes affecting stamen development, hence change the symmetry of corresponding flowers (Broholm et al., 2008; Fambrini et al., 2014b; Juntheikki-Palovaara et al., 2014; Garcês et al., 2016).

Our transgenic data show that overexpression of *CmCYC2c* in *C. lavandulifolium* leads to significant elongation in ventral ligule length of ray florets. Based on the transgenic phenotype, the high expression of *CmCYC2c* in the petals of ray and *trans*-like florets appears to be crucial for the ventral petal ligule growth. This is in consistent with the results in *Gerbera*, in which overexpression of *GhCYC3* and *GhCYC4* enhances petal ligule growth in *trans* flowers (Juntheikki-Palovaara et al., 2014). Conversely, ectopic activation of *GhCYC2, GhCYC3*, and *RAY1* can reduce ventral petal growth of corresponding ray florets (Kim et al., 2008; Juntheikki-Palovaara et al., 2014). Moreover, in sunflower and *gerbera*, overexpression of *HaCYC2c, GhCYC2*, and *GhCYC3* can cause their DF to obtain some characteristics of ray florets, while no effect is observed on the DF of *RAY1, RAY2*, and *RAY3* transgenic *Senecio* lines (Chapman et al., 2012; Juntheikki-Palovaara et al., 2014; Garcês et al., 2016). In this study, although one transgenic line generated some *trans*-like flowers, no difference was observed in DF between transgenic lines and control lines. Therefore, it appears that the CYC2 subclade proteins in Asteraceae have experienced functional diversification. Similar functional deviations have also been reported in regulation of stamen development (Juntheikki-Palovaara et al., 2014; Garcês et al., 2016). Besides promoting ventral petal length of ray florets, ectopic activation of *CmCYC2c* also statistically increased the number of ray florets per capitulum. Several studies have shown that the showy marginal flowers in Asteraceae increases the attractiveness of the inflorescence to pollinators, thereby increasing genetic diversity and fitness (Marshall and Abbott, 1984; Bremer and Anderberg, 1994; Andersson, 2008). Contrary to the single whorl of ray florets with changeless shape in the wild original species, the modern chrysanthemum cultivars are characterized by the various whorls of ray florets and the diversity of petal shape. Taking together the high expression levels of *CmCYC2c* in ray florets of the double-ray flowered chrysanthemum and the transgenic phenotypes in *C. lavandulifolium*, we can postulate that the *CmCYC2c* could be a vital regulator in evolution of modern chrysanthemum.

CYC2 clade proteins have been shown to influence vegetative and reproductive growth to produce morphological novelties in plants (Cubas et al., 1999b; Costa et al., 2005; Zhong and Kellogg, 2015). In this study, over expression of *CmCYC2c* in *C. lavandulifolium* resulted in retarded vegetative growth and reduced branching of young plantlets, which were restored gradually later, fortunately. Similar effect has been reported on many other TCP transcription factors. For example in *Gerbera*, constitutive expression of *GhCYC2* causes severe growth defects and difficulties in regeneration of transgenic shoots (Broholm et al., 2008). Similarly, ectopic activation of *CYC*-like genes in *Arabidopsis* leads to reduction in vegetative growth (Costa et al., 2005; Yang et al., 2012; Juntheikki-Palovaara et al., 2014). Thus, at least in vegetative organs, their function is conserved. However, negative regulation of bud formation or branching outgrowth is mainly documented for some other genes in CYC/TB1 group, such as *TB1* in maize (Doebley et al., 1995; Hubbard et al., 2002), *OsTB1* in rice (Takeda et al., 2003) and *BRC1* in *Arabidopsis* (Aguilar-Martínez et al., 2007). Conversely, heterologous expression of *CYC1C* in *Arabidopsis* promotes the outgrowth of lateral branches (Yang et al., 2012). Therefore, it seems like that ECE clade genes have undergone functional divergence in this content.

### The Expression of *CmCYC2f* Is Strongly Up-regulated in *CmCYC2c* Overexpressing Lines

Intriguingly, overexpression of *CmCYC2c* can strongly induce the expression of *CmCYC2f* in all transgenic lines. According to the reports in *Primulina, CYC1C* and *CYC1D* can positively autoregulate themselves and cross-regulate each other to form auto-regulatory loops, which may trigger threshold-dependent genetic witches (Yang et al., 2012). In addition, Tahtiharju et al. (2012) shows that *Gerbera* CYC2 clade proteins have the ability of interacting in yeast two-hybrid assays, and the co-regulators may target different downstream genes. Therefore, we postulate that *CmCYC2f* may work as a putative downstream target of *CmCYC2c* to induce the differentiation of specific flower in chrysanthemum. Functional analysis of *CmCYC2f* is currently underway.

## Conclusion

We show for the first time that *CYC*-like genes in chrysanthemum have undergone gene duplication and functional divergence, a condition that appears to be connected with the increased inflorescence complexity. Transcription analysis demonstrates that most of the chrysanthemum *CYC*-like genes are mainly expressed in ray florets and therefore may be functionally redundant. Of these CYC2 subclade genes, *CmCYC2c* can easily be identified as a vital gene in regulating ray floret identity. Moreover, overexpression of *CmCYC2c* is capable of regulating the growth of ray florets, whereas it is not sufficient for completely changing the floral shape. Therefore, except for *CmCYC2c*, whether the other transcription factors, such as the other *CmCYC2* members, *MYB*-domain transcription factors RADIALIS (Corley et al., 2005; Garcês et al., 2016) and DIVARICATA (Almeida et al., 1997), are involved in regulating ray floret identity in chrysanthemum awaits further study.

## Author Contributions

DH and QZ conceived and designed the experiments. XL and MS prepared the plant materials. DH and TZ performed experiments. JW, HP, and TC contributed reagents and analysis tools. DH wrote the paper.

## Conflict of Interest Statement

The authors declare that the research was conducted in the absence of any commercial or financial relationships that could be construed as a potential conflict of interest.

## Footnotes

1 http://meme-suite.org/tools/meme

## Supplementary Material

FIGURE S1 **Inflorescence developmental stages 1, 3, 4, 6, 7, 8, and 9 of *C. morifolium* ‘Mao xiangyu’ (MXY).** Stage 1 is corresponding to the developing phase of stage IV.
