# Supplementary figures and images for: Identification and Characterization of CYC-Like Genes in Regulation of Ray Floret Development in Chrysanthemum morifolium
Source: Front Plant Sci. 2016 Nov 7;7:1633. doi: 10.3389/fpls.2016.01633 (PMC5097909; doi:10.3389/fpls.2016.01633)

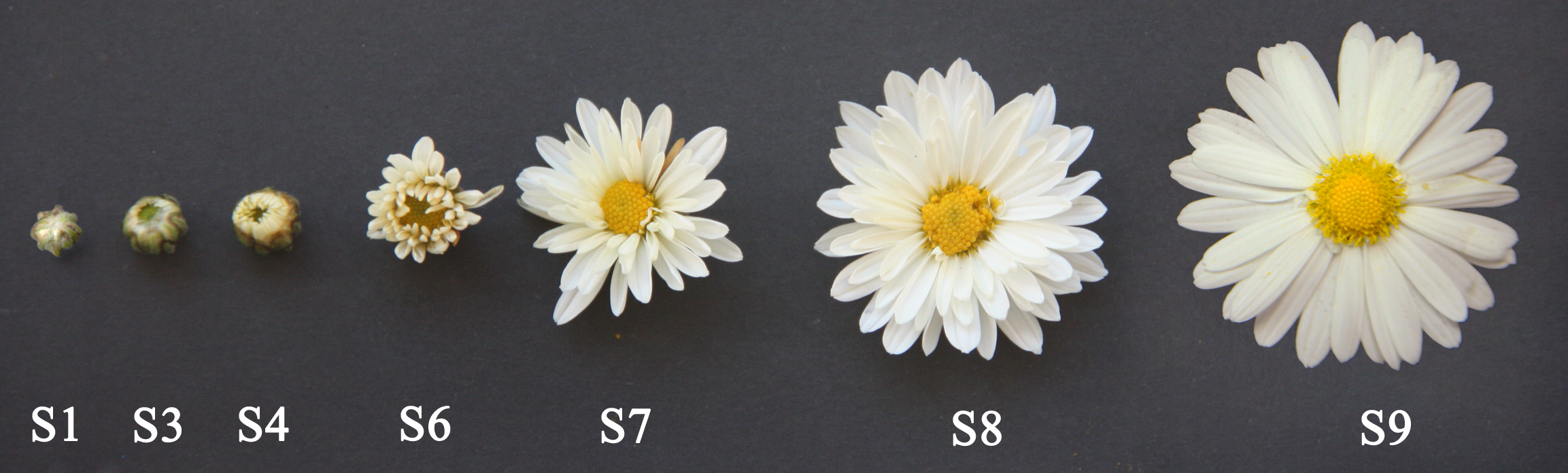

Supplement: FIGURE S1 — Inflorescence developmental stages 1, 3, 4, 6, 7, 8, and 9 of C. morifolium ‘Mao xiangyu’ (MXY). Stage 1 is corresponding to the developing phase of stage IV. [file Image_1.TIF]
