# Supplementary material for: Identification and Characterization of CYC-Like Genes in Regulation of Ray Floret Development in Chrysanthemum morifolium
Source: Front Plant Sci. 2016 Nov 7;7:1633. doi: 10.3389/fpls.2016.01633 (PMC5097909; doi:10.3389/fpls.2016.01633)
Supplement: Supplementary file 2 [file Table_1.DOCX]

**Identification and Characterization of *CYC*-like Genes in Regulation of ray flower development in *Chrysanthemum morifolium***

Di Huang^1^, Xiaowei Li^1^, Ming Sun^1^, Tengxun Zhang^1^, Huitang Pan^1^, Tangren Cheng^1^, Jia Wang^1^, Qixiang Zhang^1^*

* Correspondence: Qixiang Zhang, [zqxbjfu@126.com](mailto:zqxbjfu@126.com)

Supplementary Table 1. Primers used for CYC2 subclade genes isolation and characterization in chrysanthemum.

| Primer names | Forward sequence (5’🡪3’) | Reverse sequence (5’🡪3’) | Function of primers |
| --- | --- | --- | --- |
| Dp-F1/R1 | GCRAGCAAAACCCTWGATTGGCT | GTYCTTTCYSGCTCTYGCTCTTGC | Degenerate primers |
| *CmCYC2a*-5’GSP1 | TGCCCTTCATCTCGACCTGGTCGCCT | ATGTGAATGTTGCAGCTAGGAGCCAGT(*CmCYC2a* -3’GSP1) | 3’ and 5’ RACE of *CmCYC2* subclade genes. |
| *CmCYC2a* -5’GSP2 | CCTGGTCGCCTTTATCCATGAACACT |  |  |
| *CmCYC2b* -5’GSP1 | CGCTCTCGCTCTTGCCTCTGTCCTTGA | GGCTGGATCAGGTGGAGAAAATAGCCG (*CmCYC2b*-3’GSP1) |  |
| *CmCYC2b* -5’GSP2 | CGGCTATTTTCTCCACCTGATCCAGC |  |  |
| *CmCYC2c*-5’GSP1 | GCCCTAGACTGGTCTCTTGCTAGATTCTC | TCGAAGAACGCGATTAAGGAGCTGGT(*CmCYC2c*-3’GSP1) |  |
| *CmCYC2c*-5’GSP2 | CAACCAGCTCCTTAATCGCGTTCTTCG |  |  |
| *CmCYC2d*-5’GSP1 | CACCGGATGGGCTTACAGGGCGA | CTAGTGCTACGGATCGATGCGAAATGG(*CmCYC2d*-3’GSP1) |  |
| *CmCYC2e*-5’GSP1 | CTTTTGCCCTCAGCAGACTTTGGTGC | GGATCTCTGGTGACTCAGTCAAGGGCTG(*CmCYC2e*-3’GSP1) |  |
| *CmCYC2f*-5’GSP1 | AACCCGTTTGTCTTGTACACCCGATC | AGAACGATCGGGTGTACAAGACAAACGG(*CmCYC2f*-3’GSP1) |  |
| *CmCYC2a*-F1/R1 | AGAGCAGAGGCAAGAGCACGA | CCAATTTTGACCTCAGGATTACTTT | Primers for qRT-PCR assay in *C.morifolium* |
| *CmCYC2b*-F1/R1 | AAGAGATCAGTCAAGGACAGAGGCA | AGTTCTTTGCTGTTCCACTTGACC |  |
| *CmCYC2c*-F1/R1 | GAGCGAGAGCACGAGAAAGGACT | TCTCCTGCATTTCTTTCCAAATTAT |  |
| *CmCYC2d*-F1/R1 | ACTGTTATCGCCCTGTAAGCCCA | TTGAGAGTTTAAATCATTCGACGCA |  |
| *CmCYC2e*-F1/R1 | TGAGTCCAAGAGTGCTCATGATGAC | CGAATCGTTGAACACAGCAAGGT |  |
| *CmCYC2f*-F1/R1 | ACTATGGAACAAAATTTCTCAACGT | ACTCCCCGCACGGTCTCCT |  |
| *CmActin*-F1/R1 | CTGACAGGATGAGCAAGGAAATCAC | GAACAATGGATGGGCCAGACTC |  |
| *ClCYC2c*-utr-F1/R1 | GAGCGAGAGCACGAGAAAGGACT | AGCAATTATCATTAGCAGTTGTGGA |  |
| *ClCYC2a*-F1/R1 | AGAGCAGAGGCAAGAGCACGA | CCAATTTTGACCTCAGGATTACTTT | Primers for qRT-PCR assay in *C.lavandulifolium* |
| *ClCYC2b*-F1/R1 | AAGAGATCAGTCAAGGACAGAGGCA | AGTTCTTTGCTGTTCCACTTGACC |  |
| *ClCYC2c*-F1/R1 | GAGCGAGAGCACGAGAAAGGACT | TCTCCTGCATTTCTTTCCAAGTTAT |  |
| *ClCYC2d*-F1/R1 | ACTGTTATCGCCCTGTAAGCCCA | TTGAGAGTTTAAATCATTCGACGCA |  |
| *ClCYC2e*-F1/R1 | TGAGTCCAAGAGTGCTCATGATGAC | CGAATCGTTGAACACAGCAAGGT |  |
| *CmActin*-F1/R1 | CTGACAGGATGAGCAAGGAAATCAC | GAACAATGGATGGGCCAGACTC |  |
| *ClCYC2f*-F1/R1 | ACTATGGAACAAAATTTCTCAACGT | ACTCCCCGCACGGTCTCCT |  |
| *CmCYC2c-*F2/R2 | GGACTCTTGACCATGGTTATGTTTTCCTCAAACCCCTTTCCA | GTCAGATCTACCATGGACAACATCAGTCCAGGTCCACCAT | ORF of *CmCYC2c* for overexpression |
| 35S-CYC2c-F1/R1 | ACACGGGGGACTCTTGAC | CAGGTCCACCATTAACTCC | Primers for verifying transgenic lines |

Underlines are enzyme recognition site
