# Supplementary material for: Identification and Characterization of CYC-Like Genes in Regulation of Ray Floret Development in Chrysanthemum morifolium
Source: Front Plant Sci. 2016 Nov 7;7:1633. doi: 10.3389/fpls.2016.01633 (PMC5097909; doi:10.3389/fpls.2016.01633)
Supplement: Supplementary file 3 [file Table_2.DOCX]

**Identification and Characterization of *CYC*-like Genes in Regulation of ray flower development in *Chrysanthemum morifolium***

Di Huang^1^, Xiaowei Li^1^, Ming Sun^1^, Tengxun Zhang^1^, Huitang Pan^1^, Tangren Cheng^1^, Jia Wang^1^, Qixiang Zhang^1^*

* Correspondence: Qixiang Zhang, [zqxbjfu@126.com](mailto:zqxbjfu@126.com)

Supplementary Table 2. Developmental phases and the corresponding size (diameter) of flower heads of *C.morifolium* ‘Mao xiangyu’ (MXY).

| Phase | Characteristics | Size of capitulum (mm) |
| --- | --- | --- |
| I | Initiation of floral primordia | 1.2-1.5 |
| II | Differentiation of floral organs | 1.6-4 |
| III | Growth of floral organs | 4.1-5 |
| IV | Maturation of inflorescence | 5.1-7 |
